# Molecular docking‐guided in‐depth investigation of the biological activities and phytochemical and mineral profiles of endemic *Phlomis capitata*

**DOI:** 10.1002/jsfa.14142

**Published:** 2025-01-29

**Authors:** Ebubekir İzol

**Affiliations:** ^1^ Bee and Natural Products R&D and P&D Application and Research Center Bingöl University Bingöl Turkey

**Keywords:** *Phlomis capitata*, phytochemicals, antioxidant, antidiabetic, antiepilepsy, antiglaucoma

## Abstract

**BACKGROUND:**

*Phlomis capitata* is an endemic species of flowering aromatic and medicinal plant in the family Lamiaceae, native to regions of the Mediterranean and nearby areas. Understanding the chemical compounds present in *P. capitata* can reveal potential medicinal properties. The present study examines the quantification of bioactive phytochemicals, antioxidant capacity and enzyme inhibitory evaluation of *P. capitata* extract against key enzymes involved in the pathogenesis of Alzheimer's, diabetes mellitus, epilepsy and glaucoma for the first time. The mechanisms of enzyme inhibition activity of the predominant compounds in extract were also interpreted by molecular docking studies. Chemical characterization of the extract was performed by liquid chromatography‐tandem mass spectrometry (LC‐MS/MS) (phytochemical profile) and inductively coupled plasma‐mass spectrometry (mineral composition) analysis. Furthermore, the binding interactions of major phytochemicals with all enzymes were investigated by molecular docking studies.

**RESULTS:**

LC‐MS/MS analysis of the *P. capitata* revealed the identification of 19 compounds predominated by quinic acid (4.883 mg g^−1^), followed by chlorogenic acid (4.36 mg g^−1^), vanilic acid (3.405 mg g^−1^), naringenin (2.571 mg g^−1^) and cyranoside (1.101 mg g^−1^). It was determined that the mineral element was rich (K, Ca, Al and Mg) and did not exceed the toxicity limits. The *P. capitata* extract demonstrated remarkable antioxidant activities in the order: 2,2′‐azino‐*bis*(3‐ethylbenzothiazoline‐6‐sulfonic acid (IC_50_: 20.533 μg mL^−1^) < 2,2‐diphenyl‐1‐picrylhydrazyl (IC_50_: 23.151 μg mL^−1^) < *N*,*N*‐dimethyl‐*p*‐phenylenediamine (IC_50_: 45.221 μg mL^−1^) and cupric reducing antioxidant capacity (0.889 μg mL^−1^) < Fe^3+^ reducing (0.969 μg mL^−1^) < ferric reducing antioxidant potency (0.974 μg mL^−1^). Moreover, of all the enzyme inhibitory assays (acetylcholinesterase, butyrylcholinesterase, *α*‐amylase, *α*‐glucosidase, and human carbonic anhydrases I and II), the extract showed outstanding inhibitory activities (IC_50_ values of 3.26, 7.15, 6.15, 6.81, 15.21 and 11.93 μg mL^−1^, respectively).

**CONCLUSION:**

In summary, the findings show that *P. capitata* is a versatile raw material that can be used in the pharmaceutical, cosmetic and food industries to develop products that promote health. © 2025 The Author(s). *Journal of the Science of Food and Agriculture* published by John Wiley & Sons Ltd on behalf of Society of Chemical Industry.

## INTRODUCTION

The pharmacological activities of medicinal plants are exceedingly diverse, making them valuable resources for traditional medicine and modern pharmacological studies.^1^, ^2^, ^3^, ^4^ These plants contain a variety of chemical constituents such as alkaloids, flavonoids, terpenoids, tannins and phenolic compounds that make them generally serve as templates for drug development, contributing to treatments for a broad range of diseases. Some of the common pharmacological activities of medicinal plants include anti‐inflammatory, antioxidant and anticancer activities.^5^, ^6^, ^7^, ^8^

*Phlomis*, which has approximately 100 species, belongs to the Lamiaceae family and is primarily native to Mediterranean regions, Central Asia and parts of China. Several species of *Phlomis* have been traditionally used in herbal medicine for their therapeutic activities.^9^ It is valued for its antimicrobial, anti‐inflammatory and antioxidant activity. It has been reported to be used in the treatment of skin wounds, burns and infections.^10^, ^11^, ^12^, ^13^ These plants hold promise in treating conditions related to inflammation, infections and oxidative stress, although more scientific research is needed to fully understand their therapeutic potential.^14^, ^15^

Antioxidants are vital compounds found in various medicinal plants and they play an significant role in neutralizing harmful free radicals, which are unstable molecules that can damage cells and contribute to the development of several diseases, including cancer, cardiovascular diseases and neurodegenerative disorders.^16^, ^17^, ^18^, ^19^, ^20^ The therapeutic effects of medicinal plants with antioxidant properties have been widely recognized in traditional medicine systems for many years.^21^, ^22^, ^23^ It is known that the genus *Phlomis* contains various biologically active compounds, including flavonoids, terpenoids and phenolic acids that exhibit antioxidant, anti‐inflammatory, neuroprotective and antidiabetic properties. Minerals are metal elements that have many significant roles as well as catalysts in chemical reactions in the biological system. They are also involved in antioxidant mechanisms.^24^, ^25^ Deficiencies and excesses can cause serious diseases, whereas high concentrations of toxic ones cause poisoning and serious disorders.^26^ Their concentrations in food should be determined by reliable analysis methods.^5^ Among the most sensitive and reliable analysis techniques, inductively coupled plasma‐mass spectrometry (ICP‐MS) is the leading assay.^27^

Alzheimer's disease, diabetes, epilepsy and glaucoma are all chronic conditions that affect different systems of the human body, yet they share some underlying factors, including oxidative stress, inflammation and metabolic dysfunction.^28^, ^29^, ^30^ Enzyme inhibition plays a critical role in the management of chronic diseases such as Alzheimer's disease, diabetes, epilepsy and glaucoma. Enzyme inhibition is a key strategy in managing these diseases. Medicinal plants offer a natural and complementary approach to inhibiting specific enzymes involved in these diseases.^31^ Whether through the inhibition of acetylcholinesterase (AChE) in Alzheimer's, *α*‐glucosidase in diabetes or carbonic anhydrase in glaucoma, plant‐derived compounds have been shown to possess significant therapeutic potential.^16^ Molecular docking studies are important in drug research. Specific receptor‐ligand binding properties are pioneering in these studies. Binding affinity between receptor‐ligand is calculated. Molecular docking is an essential way to study the interactions of molecules with proteins by detecting their active sites.^2^

**Figure 1:**
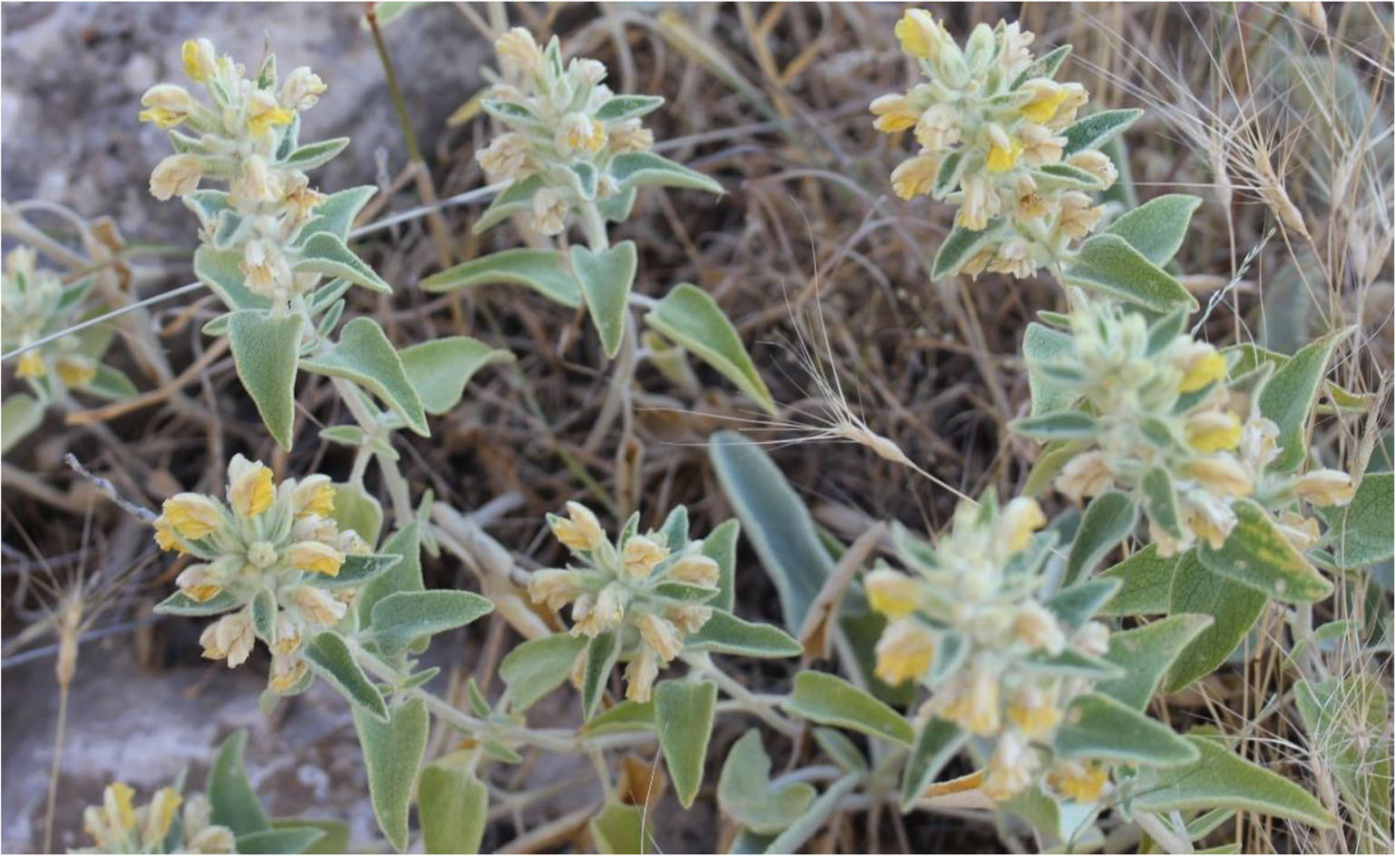
*Phlomis capitata* in the natural habitat.

Studying medicinal plants helps develop treatments with fewer side effects and may improve the quality of life for patients requiring long‐term care. Understanding the chemical compounds present in endemic *Phlomis capitata* (Fig. 1) could reveal its potential medicinal activities. The present study aimed (i) to screen and quantify the biologically active phytochemical and mineral content by liquid chromatography‐tandem mass spectrometry (LC‐MS/MS) and ICP‐MS, respectively; (ii) determine its antioxidant capacity; and (iii) perform an enzyme inhibitory evaluation of *P. capitata* extract against key enzymes [*α*‐amylase, *α*‐glucosidase, AChE, butyrylcholinesterase (BChE) and carbonic anhydrase] involved in the pathogenesis of Alzheimer's, diabetes mellitus, epilepsy and glaucoma, aiming for the first time to make molecular docking studies of major phytochemicals with respect to content.

## MATERIALS AND METHODS

### Chemicals

Chemicals and standards for LC‐MS/MS and ICP‐MS analyses were obtained from Sigma‐Aldrich (Steinheim, Germany). The suppliers of standards and chemicals for enzyme and antioxidant assays were obtained from Sigma‐Aldrich. human carbonic anhydrase I and II (hCA I and hCA II) enzymes were purified from human erythrocytes by affinity chromatography.^16^ Other enzymes were purchased commercially from Sigma‐Aldrich. Each chemical used was of analytical purity.

### Supply of plant

Plants were collected at altitudes of 1570–1630 m on stony steppe slopes between İçlikaval village and Üçpınar villages in Baskil district of Elazığ province in Turkey. They were identified in the Bingöl University herbarium and recorded in the herbarium library with the number BIN 20760.

### Preparation of the plant extract

After collection, the sample was dried in the shade and extracted with EtOH. Then, 10 g of dried plant material was macerated with 50 mL of EtOH (three times × 12 h). The solvents from the crude extract were removed and the extract was produced using a rotary evaporator at 45 °C and vacuum. The plant extract was stored in the dark at 4 °C until analysis.

### Phytochemical contents identification by LC‐MS/MS

Phytochemical identification was performed by LC‐MS/MS, which was previously validated (Yılmaz, 2020). Initially, the plant extract solution was made at a concentration of 3 mg mL^−1^. This solution was filtered through a 0.22‐μm syringe tip filter prior to transferring to LC‐MS vials. The instruments employed and data methods comprised: LCMS‐8040; autosampler model SIL‐30 AC; degasser model DGU‐20A3R; dual pump model LC‐30 AD; column oven model CTO‐10Asvp; Shimadzu, Kyoto, Japan; columns 150 mm × 2.1 mm, 2.7 μm, software LabSolutions, Shimadzu; injection volume, 5 μL; solvent flow rate, 0.5 mL min^−1^; mobile phases methanol and acetonitrile.^32^

### Determination of mineral composition by ICP‐MS

Mineral analysis was carried out by modifying the method of İzol and Turhan.^20^ The dry plant was digested in ultrapure nitric acid in a microwave extractor (MARS6 ONE TOUCH; CEM Corp., Matthews, NC, USA). The completely dissolved plants were diluted with ultrapure water using a 1% ultrapure nitric acid solution. Mineral analysis was carried out by ICP‐MS NexION® 2000 C (PerkinElmer Inc., Waltham, MA, USA). Before the analysis, calibration graphs were drawn with standards; 45Sc and 115In elements were used as internal standards, and analytical parameters are given in Table 1. Fe, Cr, Co, Mn, Ni, Cu, Zn, As, Pb, Ag, Cd, Na, Mg, Al, Se, K, Ca and V elements were analyzed by ICP‐MS and the results were determined using Syngistix, version 2.2 (PerkinElmer Inc.).^26^

**Table 1:** ICP‐MS analytical parameters

Element	Linear range	Correlation coefficient (*r* ^2^)	Limit of detection (LOD)	Limit of quantification (LOQ)
Na	0–20 (mg kg^−1^)	0.9992	0.021	0.083
Mg	0–20 (mg kg^−1^)	0.9995	0.043	0.133
Al	0–20 (mg kg^−1^)	0.9999	0.701	2.142
K	0–20 (mg kg^−1^)	0.9992	0.021	0.069
Ca	0–20 (mg kg^−1^)	0.9999	0.064	0.209
V	0–300 (μg kg^−1^)	0.9999	0.281	0.869
Cr	0–300 (μg kg^−1^)	0.9999	0.199	0.586
Mn	0–8 (mg kg^−1^)	0.9999	0.239	0.744
Fe	0–8 (mg kg^−1^)	0.9999	1.221	3.753
Co	0–300 (μg kg^−1^)	0.9999	0.228	0.803
Ni	0–300 (μg kg^−1^)	0.9999	0.314	1.054
Cu	0–300 (μg kg^−1^)	0.9997	0.611	1.899
Zn	0–8 (mg kg^−1^)	0.9999	0.320	0.992
As	0–300 (μg kg^−1^)	0.9999	0.321	0.985
Se	0–300 (μg kg^−1^)	0.9999	0.133	0.385
Ag	0–300 (μg kg^−1^)	0.9999	0.131	0.395
Cd	0–300 (μg kg^−1^)	0.9999	0.222	0.677
Pb	0–300 (μg kg^−1^)	0.9999	0.745	2.246

*Note*: LOD and LOQ concentrations of Na, Mg, K and Ca are given in mg kg^−1^; other elements are given in μg kg^−1^.

### Total phenolic and flavonoid amounts

Folin–Ciocalteu reagent was used for estimating total phenolic components in plant extract.^33^ Gallic acid was used as standard. Series of gallic acid solutions at diverse concentrations (100, 200, 300, 400, 500, 600, 700, 800, 900 and 1000 μL) were prepared by dissolving in distilled water. After that, 0.5 mL of Folin–Ciocalteu reagent and 1.5 mL of 2% Na_2_CO_3_ were added to each test tube (both standards and samples) and the mixture was shaken for 2 h. The absorbance of the mixture was determined using a UV‐visible spectrophotometer at 760 nm. The result was calculated as μg of gallic acid equivalent (GAE) per gram of extract. Distilled water was employed as a blank.^5^

A series of quercetin standard solutions at different concentrations (containing 10, 20, 30, 40 and 50 μg of quercetin) were prepared by dissolving in ethanol along with 0.1 mL (1.0 m) of CH_3_COOK (in water) and 0.1 mL of Al(NO_3_)_3_ (10%). After mixing and diluting the liquid, 4.3 mL of ethanol was added. After 40 min of regular incubation, the mixture was tested for absorbance at 415 nm against ethanol as a blank. The result was assigned as quercetin equivalent (QE).^34^

### Antioxidant activity assay

For antioxidant assays, an ethanol stock solution was made up of the plant extract at a concentration of 3 mg mL^−1^ and the assays were performed with this solution. The same concentrations of *α*‐tocopherol, butylated hydroxy anisole (BHA), Trolox and butylated hydroxytoluene (BHT) were employed as antioxidant standards.

#### Fe^3+^ reducing assay

A modified version of the approach employed by Oyaizu^35^ was used. After being pipetted at predetermined concentrations, the test tubes were vortexed. Each tube was filled with 1 mL of distilled water, vortexed and left in the dark for 10 min. Three absorbance measurements were made at 700 nm for each sample. The blank was distilled water.

#### Cupric reducing antioxidant capacity (CUPRAC) assay (Cu^2+^–Cu^+^ reducing)

The method of Apak *et al*.^36^ was applied with modification. Specific concentrations were pipetted into test tubes and mixed by vortexing. After 30 min at room conditions, absorbance was measured at 450 nm and distilled water was used as blank.

#### Ferric reducing antioxidant potency (FRAP) assay (Fe^3+^‐TPTZ reducing)

FRAP assay of the plant extract was carried out according to the method of Izol *et al*.^16^ First, test tubes were filled with standard solutions and plant stock prepared at specific concentrations (20–60 μL). These tubes were then filled with 500 μL of buffer solution and 2250 μL of FRAP and FeCl_3_ solutions were spiked to each tube. The tubes were then vortexed and kept in the dark for 30 min and absorbance was recorded at 593 nm.

#### 2,2‐Diphenyl‐1‐picrylhydrazyl (DPPH)· scavenging assay

The DPPH assay of the plant extract was performed by the method of Blois.^37^ Here, 1 mm DPPH solution was utilized as oxidant. The absorbance of the radical and ethanol control solution was adjusted to 1 ± 0.025 nm before analysis. Different concentrations of the extract (20–60 μg mL^−1^) were pipetted into test tubes, vortexed and then left in the absence of light for 30 min under room conditions. Finally, the absorbance at 517 nm was recorded against blank (ethanol).^3^

#### 2,2′‐Azino‐*bis*(3‐ethylbenzothiazoline‐6‐sulfonic acid (ABTS)·^+^ scavenging assay

The ABTS assay was conducted by modifying the method of Re *et al*.^38^ Prior to analysis, the absorbance of the control solution containing phosphate buffer and ABTS·^+^ solution was adjusted to 1 ± 0.025 nm. Specific concentrations of sample (20–60 μg mL^−1^), 0.1 m phosphate buffer and 2 mm ABTS·^+^ solution were pipetted into test tubes. After mixing, they were kept for 30 min under room conditions in the dark. Finally, absorbance at 734 nm was registered using phosphate buffer as blank.

#### *N*,*N*‐dimethyl‐*p*‐phenylenediamine (DMPD)·^+^ scavenging assay

The DMPD assay was conducted according to the method of Gülçin *et al*.^39^ First, 0.01 m DMPD·^+^ reagent was prepared by mixing 1 mL of 0.1 m DMPD solution and 0.2 mL of 0.05 m FeCl_3_ solution to (pH 5.25) 100 mL of 0.1 m sodium acetate buffer. Before analysis, the absorbance of distilled water and DMPD·^+^ reagent control solution was adjusted to 1 ± 0.025 nm. Distilled water, DMPD·^+^ reagent and sample at specific concentrations (20–60 μg mL^−1^) were pipetted into the test tube and mixed. This was then incubated for 50 min at room conditions. Finally, absorbance at 505 nm was recorded with buffer used as blank.

### Enzyme inhibition activity assay

#### AChE and BChE enzyme inhibition activity assay

In these methods, enzyme inhibition was determined by thiocholine. Thiocholine and 5,5′‐dithio‐bis‐(2‐nitrobenzoic acid) (DTNB) combine to form 5‐thio‐2‐nitrobenzoic acid, which is yellow and absorbs light at 412 nm.^40^ The IC_50_ value was used to measure enzyme inhibition. Accordingly, the test tube was filled with buffer, purified water, DTNB solution, enzyme solution, inhibitor and substrate (acetyl and butyrylcholine iodate) in specific amounts. The absorbance was then monitored at 412 nm for 3 min. The concentration *versus* percentage enzyme activity was then plotted, and the IC_50_ value was calculated using the graph equation.

#### *α*‐Amylase enzyme inhibition activity assay

This was determined according to the methodology of Xiao *et al*.^41^ The enzyme was obtained from a commercial supplier. Different concentrations of the inhibitor, starch, buffer and enzyme were added to the cuvette. The procedure was used to calculate various sample concentrations, and the absorbance readings at 580 nm were used to calculate the IC_50_.

#### *α*‐Glycosidase enzyme inhibition activity assay

This was determined according to the method of Tao *et al*.^42^ The cuvette was filled with predetermined amounts of buffer, purified water, enzyme solution, inhibitor and *p*‐nitrophenyl glucopyranoside. Different concentrations of stock solution of the plant extract were pipetted to determine the IC_50_ value. After 3 min, absorbance values were determined at 405 nm and IC_50_ values were obtained.

#### hCA I and hCA II isoenzymes inhibition activity assay

The esterase activity approach was used to determine enzyme inhibition by calculating IC_50_ values. Accordingly, different concentrations of plant extract stocks were pipetted from stocks prepared with dimethyl sulfoxide, and the absorbance was monitored at 348 nm for 3 min. The enzyme activity was plotted as a concentration percentage of the graph, and IC_50_ values were calculated using the graph equation.^22^

### Molecular docking

Molecular docking was accomplished using the AutoDock Vina tool^43^ and UCSF Chimera software.^44^ For molecular docking studies, bioactive phytochemicals (cyranoside, chlorogenic acid, naringenin, quinic acid and vanillic acid) were docked with target enzymes (cholinesterase, *α*‐glycosidase, *α*‐amylase, hCA I and hCA II) using AutoDock Vina. After docking analysis, the results were recorded. Protein–ligand interactions were evaluated in BIOVIA Discovery Studio (https://www.3ds.com/products/biovia/discovery-studio) and amino acids were labeled. Finally, both 2D and 3D structures of the protein–ligand interface were revealed.^2^, ^45^

### Statistical analysis

Statistical analyses were accomplished using SPSS (IBM Corp., Armonk, NY, USA). Results are reported as the mean ± SD from three independent trials (*n* = 3). One‐way analysis of variance was applied for differences and Tukey's post‐hoc test was used for comparisons. *P* < 0.05 was considered statistically significant.

## RESULTS AND DISCUSSION

### LC‐MS/MS analysis of *P. capitata*

Phytochemicals are naturally occurring substances identified in plants that are involved in several biological activities. Although they are not essential nutrients, they play a crucial role in plant defense mechanisms and human health. Phytochemicals have gained attention for their potential therapeutic properties, including anticancer, anti‐inflammatory, antioxidant, antimicrobial and immune‐boosting effects.

**Figure 2:**
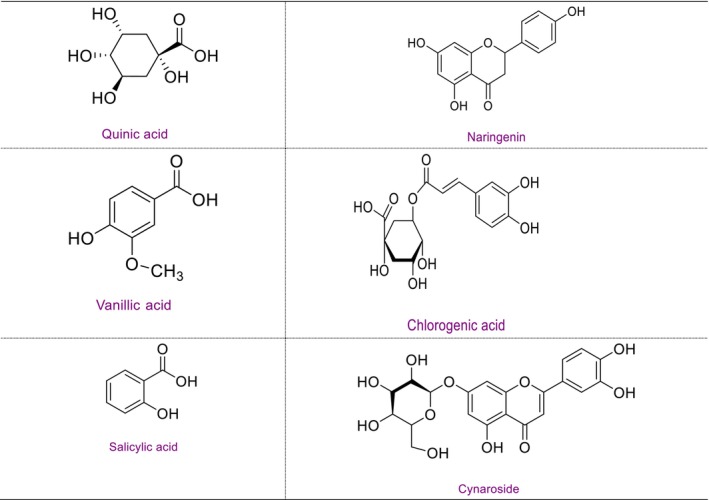
Molecular structure of predominant phytochemicals found in *Phlomis capitata*

The phytochemical investigation of *P. capitata*, a species from the Lamiaceae family, involves analyzing its chemical constituents to identify bioactive compounds that could be responsible for its traditional medicinal uses. Detailed profiles of phytochemical content of *P. capitata* ethanol extract are provided in Table 2. LC‐MS/MS profiling confirmed the presence of 19 compounds through screening of 53 phytochemicals from ethanol extract. Phytochemicals, such as quinic acid (4.883 mg g^−1^), chlorogenic acid (4.36 mg g^−1^), vanillic acid (3.405 mg g^−1^), naringenin (2.571 mg g^−1^) and cyranoside (1.101 mg g^−1^), were identified from ethanol extract of the plant. The molecular structures of these phytochemicals are shown in Figure 2.

**Table 2:** Phytochemical content results of *P. capitata* (mg analyte g^−1^ extract)

No.	Secondary metabolites	RT	MI (*m*/*z*)	FI (*m*/*z*)	*r* ^*2*^	LOD/LOQ (μg L^−1^)	*Phlomis capitata*
1	**Quinic acid**	3.0	190.8	93.0	0.996	25.7/33.3	**4.883**
2	Fumaric aid	3.9	115.2	40.9	0.995	135.7/167.9	–
3	Aconitic acid	4.0	172.8	129.0	0.971	16.4/31.4	–
4	Gallic acid	4.4	168.8	79.0	0.999	13.2/17.0	–
5	Epigallocatechin	6.7	304.8	219.0	0.998	237.5/265.9	–
6	Protocatechuic acid	6.8	152.8	108.0	0.957	21.9/38.6	0.077
7	Catechin	7.4	288.8	203.1	0.999	55.0/78.0	–
8	Gentisic acid	8.3	152.8	109.0	0.997	18.5/28.2	–
9	**Chlorogenic acid**	8.4	353.0	85.0	0.995	13.1/17.6	**4.36**
10	Protocatechuic aldehyde	8.5	137.2	92.0	0.996	15.4/22.2	0.146
11	Tannic acid	9.2	182.8	78.0	0.999	15.3/22.7	–
12	Epigallocatechin gallate	9.4	457.0	305.1	0.999	61.0/86.0	–
13	Cynarin	9.8	515.0	191.0	0.999	5.8/9.4	–
14	4‐OH benzoic acid	10.5	137.2	65.0	0.999	68.4/88.1	–
15	Epicatechin	11.6	289.0	203.0	0.996	139.6/161.6	–
16	**Vanilic acid**	11.8	166.8	108.0	0.999	141.9/164.9	**3.405**
17	Caffeic acid	12.1	179.0	134.0	0.999	7.7/9.5	0.034
18	Syringic acid	12.6	196.8	166.9	0.998	82.3/104.5	–
19	Vanillin	13.9	153.1	125.0	0.996	24.5/30.4	0.179
20	Syringic aldehyde	14.6	181.0	151.1	0.999	19.7/28.0	–
21	Daidzin	15.2	417.1	199.0	0.996	7.0/9.5	–
22	Epicatechin gallate	15.5	441.0	289.0	0.997	19.5/28.5	–
23	Piceid	17.2	391.0	135/106.9	0.999	13.8/17.8	–
24	*p*‐Coumaric acid	17.8	163.0	93.0	0.999	25.9/34.9	0.037
25	Ferulic acid‐D3‐IS	18.8	196.2	152.1	NA	NA	NA
26	Ferulic acid	18.8	192.8	149.0	0.999	11.8/15.6	–
27	Sinapic acid	18.9	222.8	193.0	0.999	65.2/82.3	–
28	Coumarin	20.9	146.9	103.1	0.999	214.2/247.3	–
29	Salicylic acid	21.8	137.2	65.0	0.999	6.0/8.3	0.312
30	**Cyranoside**	23.7	447.0	284.0	0.997	12.1/16.0	**1.101**
31	Miquelianin	24.1	477.0	150.9	0.999	10.6/14.7	–
32	Rutin‐D3‐IS	25.5	612.2	304.1	NA	NA	NA
33	Rutin	25.6	608.9	301.0	0.999	15.7/22.7	–
34	isoquercitrin	25.6	463.0	271.0	0.998	8.7/13.5	0.043
35	Hesperidin	25.8	611.2	449.0	0.999	19.0/26.0	–
36	*o*‐Coumaric acid	26.1	162.8	93.0	0.999	31.8/40.4	–
37	Genistin	26.3	431.0	239.0	0.991	14.9/21.7	–
38	Rosmarinic acid	26.6	359.0	197.0	0.999	16.2/21.2	–
39	Ellagic acid	27.6	301.0	284.0	0.999	56.9/71.0	–
40	Cosmosiin	28.2	431.0	269.0	0.998	6.3/9.2	0.474
41	Quercitrin	29.8	447.0	301.0	0.999	4.8/6.4	–
42	Astragalin	30.4	447.0	255.0	0.999	6.6/8.2	–
43	Nicotiflorin	30.6	592.9	255.0/284.0	0.999	11.9/16.7	–
44	Fisetin	30.6	285.0	163.0	0.999	10.1/12.7	–
45	Daidzein	34.0	253.0	223.0	0.999	9.8/11.6	–
46	Quercetin‐D3‐IS	35.6	304.0	275.9	NA	NA	NA
47	Quercetin	35.7	301.0	272.9	0.999	15.5/19.0	–
48	**Naringenin**	35.9	270.9	119.0	0.999	2.6/3.9	**2.571**
49	Hesperetin	36.7	301.0	136.0/286.0	0.999	7.1/9.1	0.094
50	Luteolin	36.7	284.8	151.0/175.0	0.999	2.6/4.1	0.517
51	Genistein	36.9	269.0	135.0	0.999	3.7/5.3	–
52	Kaempferol	37.9	285.0	239.0	0.999	10.2/15.4	0.008
53	Apigenin	38.2	268.8	151.0/149.0	0.998	1.3/2.0	0.172
54	Amentoflavone	39.7	537.0	417.0	0.992	2.8/5.1	–
55	Chrysin	40.5	252.8	145.0/119.0	0.999	1.5/2.8	–
56	Acacetin	40.7	283.0	239.0	0.997	1.5/2.5	0.061

*Note*: Bold value represent ingredients that are present in high concentration.

Abbreviations: −, not detected; D3, deuterium isotope 3; FI (*m/z*), fragment ions; IS, internal standard; LOD/LOQ (μg L^−1^), limit of detection/quantification; MI (*m/z*), molecular ions of the standard analytes (*m*/*z* ratio); N., numbers; NA, not applicable; RT, retention time; *r* ^*2*^, coefficient of determination.

**Figure 3:**
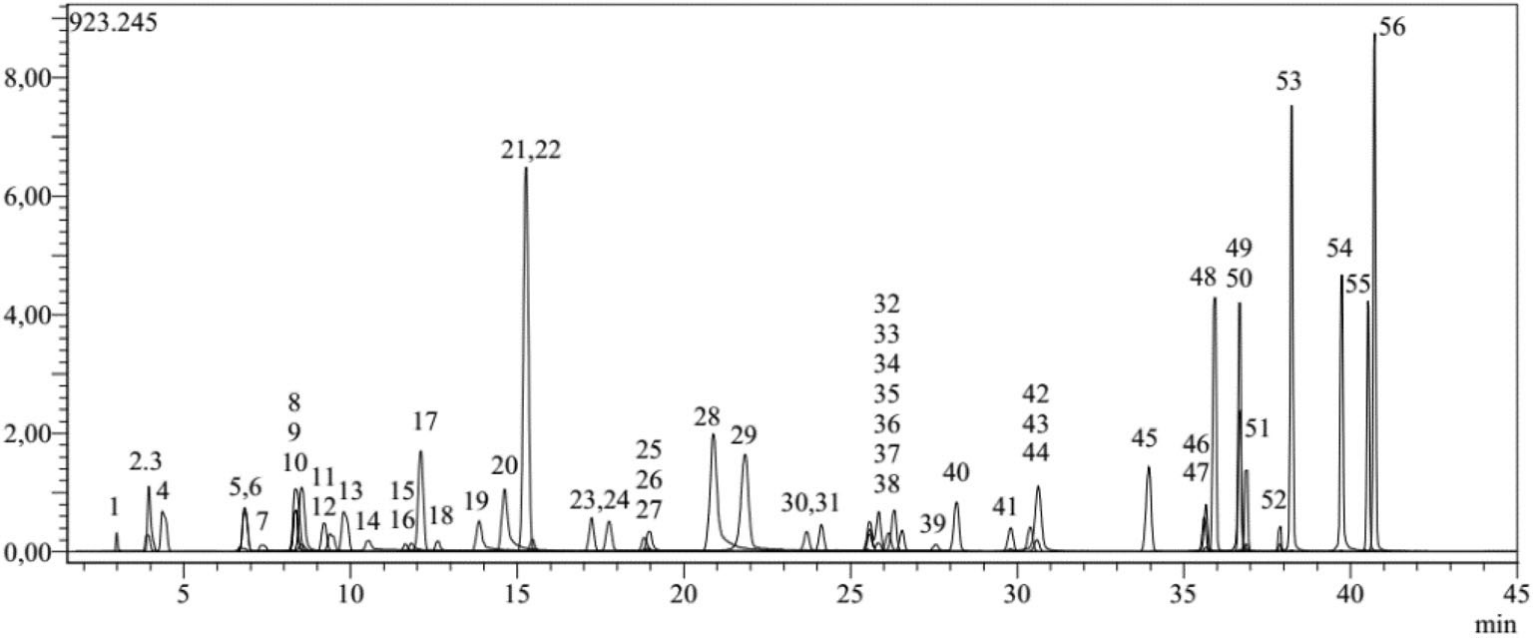
LC‐MS/MS standard chromatogram.

**Figure 4:**
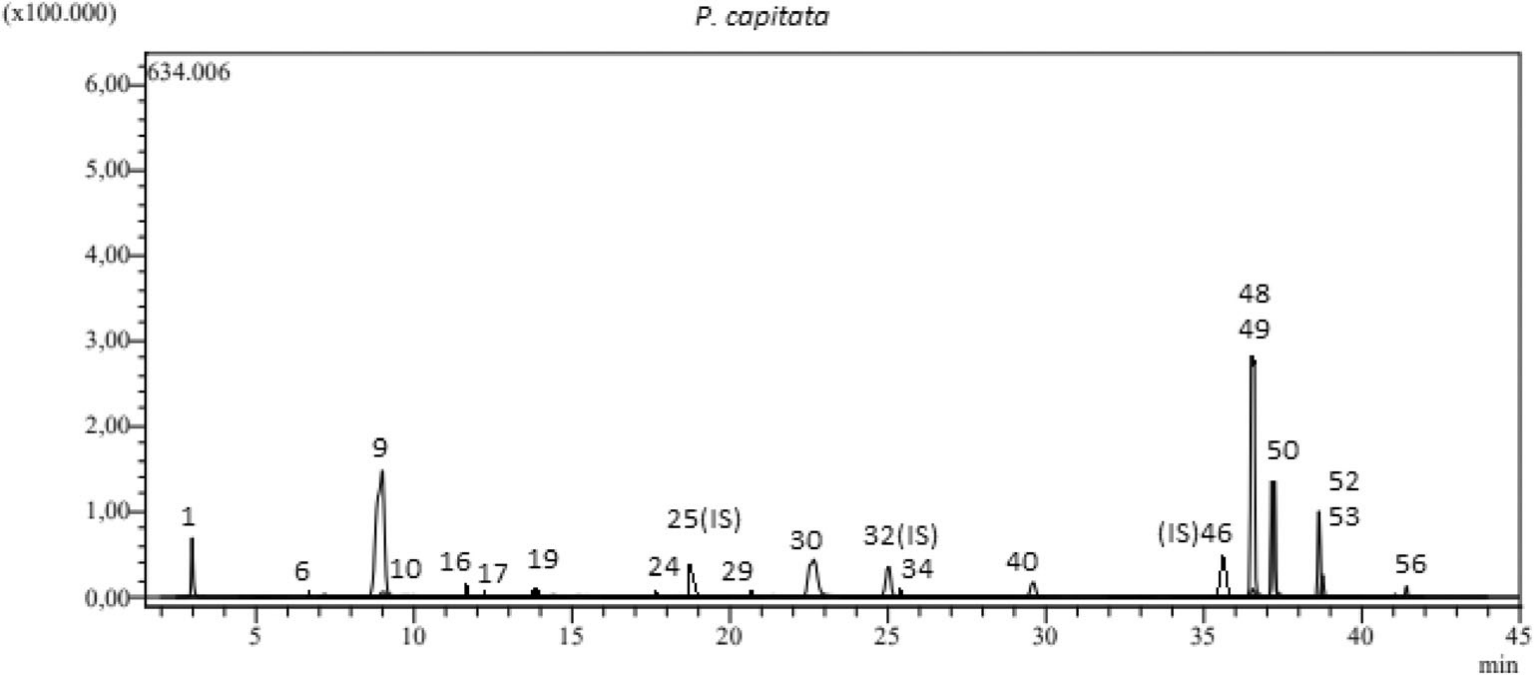
LC‐MS/MS chromatogram of *Phlomis capitata*.

Chromatograms of *P. capitata* and standards are presented in Figs 3 and 4

Naringenin, luteolin, eriodictyol, apigenin and chryseriol are reported as the most commonly occurring flavonoids in the *Phlomis* species.^46^

### ICP‐MS analysis of *P. capitata*

The minerals Fe, Se, Co, Zn, Ni, As, Cu, Pb, Ag, Cd, Na, Mn, Mg, Cr, Al, K, Ca and V were quantitatively determined by ICP‐MS of *P. capitata*. The results are reported in Table 3.

**Table 3:** Mineral concentrations of *Phlomis capitata*

Sample	Na (mg kg^−1^)	Mg (mg kg^−1^)	Al (mg kg^−1^)	K (mg kg^−1^)	Ca (mg kg^−1^)	V (mg kg^−1^)
*Phlomis capitata*	160.3 ± 5.2	1386.9 ± 11.7	1549.0 ± 21.3	9855.1 ± 97.1	5647.32	3.8 ± 0.05

Sample	Cr (mg kg^−1^)	Mn (mg kg^−1^)	Fe (mg kg^−1^)	Co (mg kg^−1^)	Ni (mg kg^−1^)	Cu (mg kg^−1^)
*Phlomis capitata*	1.8 ± 0.07	60.2 ± 2.14	1235.1 ± 33.5	0.7 ± 0.001	1.3 ± 0.03	5.0 ± 0.11

Sample	Zn (mg kg^−1^)	As (μg kg^−1^)	Ag (μg kg^−1^)	Cd (μg kg^−1^)	Pb (μg kg^−1^)	Se (μg kg^−1^)
*Phlomis capitata*	22.9 ± 1.13	0.5 ± 0.007	18.1 ± 2.21	63.4 ± 3.15	1.6 ± 0.02	< LOD

*Note*: LOD, limit of detection. Results are reported as the mean ± SD of three parallel calculations.

The highest mineral concentration in *P. capitata* was K (9855 mg kg^−1^) and the lowest concentration was As (0.5 μg kg^−1^). Because this is the inaugural investigation into the mineral concentration of this plant, the findings cannot be juxtaposed with those of previous studies.

Minerals are very significant for living systems and play a role in the formation of many active chemicals and act as catalysts in many reactions. In most antioxidant methods, the mechanisms are realized with minerals. However, high concentrations of some minerals have a toxic effect. For this reason, metal analyses should be carried out regularly in the foods consumed, and those foods should not be consumed if toxic properties are detected.^47^

In a previous study conducted, the Al concentration range in various plant species was found to be in the range 30–1424 mg kg^−1^.^48^ In the present study, Al was determined as 1549 mg kg^−1^. It was determined that the Al concentration was higher than the study. Cr acts as a cofactor in the synthesis of cholesterol and insulin hormone. In different studies, it has been reported that Cr concentration in plants varies between 0.15 and 35 mg kg^−1^.^49^ In the present study, Cr was found to be 4.8 mg kg^−1^. This result was found to be consistent with the previous studies. According to World Health Organization (WHO) data, the toxicity limits of Cd, Cr, As and Pb metals in raw plants were determined as 0.3, 2, 5 and 10 mg kg^−1^, respectively.^50^ In the present study, concentrations of Cd at 63.4 μg kg^−1^, Cr at 1.8 mg kg^−1^, As at 0.5 μg kg^−1^ and Pb at 1.6 μg kg^−1^ were detected, none of which exceeded established toxicity thresholds. The maximum Ni concentration in plants was determined by WHO as 1.63 mg kg^−1^. In another study, it was determined to be in the range 24–4740 (μg kg^−1^).^47^ In the present study, it was found to be 1.3 mg kg^−1^, and this value did not exceed the toxicity limit and was found to be compatible with other studies. Cu is a micronutrient element for plants and shows toxic properties at high concentrations. In one study, it was detected in plants in the range 0.2–24 mg kg^−1^.^49^ In the present study, it was determined as 5 mg kg^−1^, and this value did not exceed the toxicity limit. It was determined that *P. capitata* is a very rich source of K and contains high concentrations of Ca, Al, Mg and Fe minerals. The concentrations of toxic heavy metals did not exceed the toxicity limits.

### Antioxidant activity analysis

The need for a thorough assessment of the antioxidant capacity of the ethanol extract of *P. capitata* has been fueled by the lack of scientific data regarding its potential antioxidant capacities. Eight bioassays were carried out to assess the antioxidant capacity of the *P. capitata* extract. These assays included, reducing power (Fe^3+^, FRAP and CUPRAC) and free‐radical scavenging (DMPD, DPPH and ABTS). These findings are presented in Tables 4 and 5. As demonstrated in Table 4, the ethanol extract of *P. capitata* showed the highest scavenging activity against DMPD (IC_50_ of 45.221 μg mL^−1^), DPPH (IC_50_ of 23.151 μg mL^−1^) and ABTS (IC _50_ of 20.533 μg mL^−1^).

**Table 4:** Results of DPPH·, ABTS·^+^ and DMPD·^+^ scavenging assays of *Phlomis capitata*

Standards and sample		DPPH^·^ (λ_517_)		ABTS^·+^ (λ_734_)		DMPD^·+^ (λ_505_)	
		IC_50_	*r* ^2^	IC_50_	*r* ^2^	IC_50_	*r* ^2^
Standards	BHA	21.613 ± 1.007	0.9747	11.264 ± 1.012	0.9804	40.120 ± 1.085	0.9936
	BHT	19.451 ± 2.021	0.9882	11.940 ± 1.0 58	0.9938	37.251 ± 1.005	0.9553
	*α*‐Tokoferol	16.547 ± 1.001	0.9707	16.113 ± 1.154	0.9754	41.521 ± 1.721	0.9421
	Trolox	15.465 ± 1.013	0.9958	10.727 ± 1.013	0.9675	33.657 ± 2.002	0.9777
	*Phlomis capitata*	23.151 ± 1.012	0.9825	20.533 ± 1.003	0.9889	45.221 ± 2.112	0.9891

*Note*: All values are the mean of three parallel measurements (n = 3) and are presented as the mean ± SD (*P* < 0.05 is considered statistically significant). Absorbance concentrations were taken as 40 μg mL^−1^. The IC_50_ concentrations are μg mL^−1^.

**Table 5:** Fe^3+^, CUPRAC and FRAP‐reducing results of *Phlomis capitata*

Standards and sample		Fe^3+^ reducing		Cu^2+^ reducing		Fe^3+^‐TPTZ reducing	
		λ_700_	*r* ^2^	λ_450_	*r* ^2^	λ_593_	*r* ^2^
Standards	BHA	1.709 ± 0.079	0.9821	1.173 ± 0.112	0.9758	1.030 ± 0.014	0.9815
	BHT	0.818 ± 0.095	0.9712	0.818 ± 0.095	0.9806	0.699 ± 0.092	0.9798
	*α*‐Tokoferol	1.470 ± 0.028	0.9513	2.255 ± 0.167	0.9597	1.526 ± 0.041	0.9682
	Trolox	1.848 ± 0.049	0.9921	0.743 ± 0.048	0.9834	0.994 ± 0.018	0.9892
	*Phlomis capitata*	0.969 ± 0.024	0.9911	0.889 ± 0.012	0.9901	0.974 ± 0.043	0.9891

*Note*: All values are the mean of three parallel measurements (n = 3) and are presented as the mean ± SD (*P* < 0.05 is considered statistically significant). Concentrations are given in 40 μg mL^−1^.

The results of Fe^3+^, CUPRAC and FRAP‐reducing assays of *P. capitata* are given in Table 5.

The reducing capacity of the substances to donate an electron and consequently behave as reducing agents is mainly evaluated through two commonly used methodologies, including FRAP (ferric ion) and CUPRAC (cupric ion) assays. In the present study, the ethanol extract of *P. capitata* exhibited the highest Fe^3+^ (129.55 mg TE g^−1^)‐ and Cu^2+^ (229.37 mg TE g^−1^)‐reducing potentials. In the Fe^+3^ reducing assay, *P. capitata* showed higher activity than the BHT standard antioxidant and lower activity than BHA, *α*‐tocopherol and Trolox standards. In this assay, antioxidant properties were ranked as Trolox > BHA > *α*‐tocopherol > *P. capitata* > BHT. In the CUPRAC method, it was determined that the plant showed higher antioxidant properties in BHT and Trolox standards and lower antioxidant properties in BHA and *α*‐tocopherol. The ranking of antioxidant activity was found to be *α*‐tocopherol > BHA > *P. capitata* > BHT > Trolox. In the FRAP assay, it was detected that the plant showed antioxidant properties higher than the BHT standard, close to Trolox and lower than the other two standards. The order of antioxidant activity was determined as *α*‐tocopherol > BHA > Trolox > *P. capitata* > BHT.

The results of the total phenolic and flavonoid content of *P. Capitata* are shown in Table 6.

**Table 6:** Results of total phenolic and flavonoid content of plant extract

Sample	Total phenolic contents (mg GAE g^−1^)	Total flavonoid contents (mg QE g^−1^)
*Phlomis capitata*	54.971 ± 2.211	21.896 ± 0.979

*Note*: The values given are the three parallel measurements' mean ± SD.

Abbreviations: GAE, gallic acid equivalents; QE, quercetin equivalents.

Total phenolic and total flavonoid content of *P. capitata* plant was 54.971 mg GAE g^−1^ and 21.896 mg QE g^−1^, respectively.

Because the antioxidant properties of *P. capitata* have not yet been studied, the results were compared with other *Phlomis* species. In one study, antioxidant activities of *Phlomis pungens* were determined by DPPH, CUPRAC, FRAP and ABTS assays. *P. capitata* (IC_50_: 23.151 μg mL^−1^) showed higher DPPH radical scavenging activity than *P. pungens* (IC_50_: 64 μg mL^−1^). Total phenolic content was found to be close to each other in the two plants.^51^

In the study in which the antioxidant properties of *Phlomis nissolii* were determined, the ethyl acetate and methanol extracts of *P. nissolii* had greater levels of total phenolics than *P. pungens* var. pungens. Conversely, *P. pungens* var. pungens was found to contain higher levels of total flavonoids, with the exception of the ethyl acetate fraction. A comparable data profile was also noted for this plant's total flavanol content. The *P. nissolii* extract with the lowest phenolic content was ethyl acetate extract. However, the amounts of phenolic compounds in the water and methanol extracts were almost equal.^9^ In the two studies, it was observed that the antioxidant properties of different *Phlomis* species were parallel. In a study, the DPPH assay was applied to determine the antioxidant properties of *Phlomis angustissima* and *Phlomis fruticosa*, which are endemic to Turkey. Extracts of leaves and flower parts of the plants in different solvents were investigated. It was determined that the plant showing the highest DPPH activity was the aqueous extract of the flower part of *P. angustissima* (IC_50_: 37.08 mg kg^−1^). It was also determined that *Phlomis* species consumed as tea have good antioxidant properties.^52^ Because *P. capitata* was also found to have good antioxidant properties, the results for these three species were compatible with each other.

In the study conducted in 2023, the antioxidant properties of *Phlomis tuberosa* were determined by the methods employed in the present study. The total phenolic content of methanol and water extracts was 70.5 and 28 mg GAE g^−1^, respectively, and the total flavonoid content was 116.7 and 160.8 mg QE g^−1^, respectively. In general, the phenolic content was expected to be higher than the flavonoid content, but the flavonoid content was very high in the previous study.^12^ In *P. capitata*, it was observed that the total phenolic content was lower than the methanol extract of *P. tuberosa* and higher than the water extract, whereas the total flavonoid content was low. In the same study, Fe^3+^ reducing, CUPRAC, FRAP, DPPH, ABTS and DMPD assays showed that the plant showed lower activity than standard antioxidants. However, it was stated that it showed activity close to some standards and therefore its antioxidant properties were good.^12^ It was determined that *P. capitata* showed more activity in antioxidant standards in some assays. Therefore, it was determined that *P. capitata* showed better antioxidant activity than *P. tuberosa*.

### Enzyme inhibitory effects of *P. capitata*

*Phlomis* species is mainly known for its therapeutic properties and has been traditionally used in different cultures for treatment of various diseases such as diabetes, cognitive disorders and respiratory issues. Its bioactive compounds, including flavonoids and phenolic acids, have been shown to contribute to its superior pharmacological properties, particularly as enzyme inhibitors. The present study has also explored the inhibitory effects of *P. capitata* on five key enzymes: *α*‐amylase, *α*‐glucosidase, AChE, BChE and human carbonic anhydrases (hCA I and II). Table 7 demonstrates the enzyme inhibitory action of ethanol extract of *P. capitata* against these enzymes.

**Table 7:** IC_50_ of hCA I, hCA II, AChE, BChE, *α*‐glycosidase and *α*‐amylase inhibition of *Phlomis capitata*

Sample	hCA I		hCA II		AChE		BchE		*α*‐ Glycosidase		*α*‐ Amylase	
	IC_50_	*r* ^2^	IC_50_	*r* ^2^	IC_50_	*r* ^2^	IC_50_	*r* ^2^	IC_50_	*r* ^2^	IC_50_	*r* ^2^
*Phlomis capitata*	15.21	0.9912	11.93	0.9769	3.26	0.9778	7.15	0.9794	5.71	0.9796	4.12	0.9871
Acetazolamide	3.12	0.9895	4.77	0.9895	–	–	–	–	–	–	–	–
Tacrine	–	–	–	–	2.12	0.9981	5.21	0.9812	–	–	–	–
Acarbose	–	–	–	–	–	–	–	–	6.81	0.9980	6.15	0.9873

*Note*: Concentrations are given as (μg mL^−1^). Acarbose was employed as the standard inhibitor for *α*‐glycosidase and *α*‐amylase enzymes; acetazolamide was utilized as the standard inhibitor for hCA I and hCA II enzymes; and tacrine was used.

Amylase plays a critical role in carbohydrate digestion through breaking down starches into mono‐ and disaccharides. Inhibiting amylase could be beneficial for treatment of hyperglycemia, especially in diabetic patients. Studies have revealed that extracts of *P. capitata* demonstrates significant amylase inhibitory activity. The amylase inhibitory activity of *P. capitata* is attributed to its polyphenolic compounds, which may bind to the active site of the enzyme, thereby preventing substrate access. In the present study, ethanol extract of the plant showed great inhibitory effect against *α*‐amylase with an IC_50_ of 4.12 μg mL^−1^ which appears to be greater than that of standard drug acarbose (IC_50_ of 6.81 μg mL^−1^). Because *P. capitata* is a poorly studied endemic species, the enzyme inhibitory effects of the plant will be compared with different *Phlomis* species. A study by Stojković *et al*.^53^ examined the potential inhibitory effects of *P. fruticosa* L. on *α*‐amylase activity, revealing that the extract significantly reduced enzyme activity, suggesting its potential for glycemic control in management of diabetes. Another study by Eruygur *et al*.^6^ highlighted the antidiabetic properties of various medicinal *Phlomis* species, by demonstrating the ability to inhibit both *α*‐amylase and *α*‐glucosidase, supporting their use in traditional medicine.

One of the critical enzymes involved in carbohydrate metabolism is *α*‐glucosidase, which catalyzes the hydrolysis of *α*‐glucosidic linkages in carbohydrates, leading to the absorption of glucose. Inhibiting *α*‐glucosidase may prohibit absorption of glucose, aiding the management of hyperglycemia in diabetic patients. In the present study, it was found that ethanol extract of the plant showed great inhibitory effect against *α*‐glucosidase with an IC_50_ of 5.71 μg mL^−1^. A study carried out by Rasheed *et al*.^54^ assessed the *α*‐glucosidase inhibitory activity of *Phlomis stewartii* extracts. The results demonstrated a significant dose‐dependent inhibition of *α*‐glucosidase, suggesting the potential of the plant extract in managing diabetes. The researchers attributed this effect to the presence of flavonoids and other phenolic compounds in the extract, which were shown to have strong antioxidant properties and potential antidiabetic effects. Another study by El‐Azab *et al*.^55^ compared the *α*‐glucosidase inhibitory effects of *Phlomis aurea* with known antidiabetic drugs. It was found that *P. aurea* exhibited comparable inhibition levels to acarbose, a commonly prescribed *α*‐glucosidase inhibitor. The findings highlight the potential of *Phlomis* species as a natural alternative for managing diabetes. A study by Kondeva‐Burdina *et al*.^56^ successfully evaluated the inhibitory effects on *α*‐glucosidase. Certain compounds were found to exhibit strong inhibitory activity, suggesting that these phytochemicals could be developed into functional foods or supplements for diabetes management.

AChE and BChE are two important enzymes that participate in neurotransmitter regulation. Inhibition of these enzymes has been recognized as a therapeutic strategy in managing Alzheimer's disease and other neurodegenerative disorders because they enhance availability of acetylcholine in the synaptic cleft. The present study has revealed that AChE and BChE activity were inhibited by the plant extract with an IC_50_ of 3.26 and 7.15 μg mL^−1^, respectively. In a different study, Sarıkurkcu *et al*.^9^ evaluated the AChE inhibitory activity of *P. nissolii* and *P. pungens* var. pungens, demonstrating significant enzyme inhibition, which suggests their potential use in treating cognitive impairments. In another study carried out by the same group, the ethyl acetate extract of *P. armeniaca* showed the highest activity against BChE (4.579 mg GALAEs g^−1^ extract).^57^

Human carbonic anhydrases (hCA I and II) are enzymes that catalyze the reversible hydration of carbon dioxide and are essential for various physiological processes, including acid–base balance and respiratory function. The inhibition of these enzymes has therapeutic potential in treating conditions such as glaucoma, epilepsy and certain respiratory disorders. The inhibitory effect of *P. capitata* on hCA I and II is attributed to its high‐affinity compounds that can bind to the active site of the enzyme, disrupting its catalytic function. This study also assessed the inhibitory eggect of *P. capitata* extract against hCA I and hCA II. The results demonstrated that the extract nhibited the activity of the enzymes with an IC_50_ of 15.20 and 11.93 μg mL^−1^, respectively. Güven *et al*.^12^ evaluated the inhibitory effects of herbal extracts on human carbonic anhydrases, highlighting *P. tuberosa* as a significant inhibitor, suggesting its potential in treating disorders related to carbonic anhydrase activity. Sarıkürkçü *et al*.^58^ further emphasized the emerging role of carbonic anhydrase inhibitors in therapeutic applications, including those derived from natural sources such as *Phlomis leucophracta*.

One study reported *α*‐glycosidase, AChE and hCA II enzyme inhibitions by *P. tuberosa*. It was observed that *α*‐glycosidase was inhibited with an IC_50_ of 2.21 μg mL^−1^ in the presence of showing a higher inhibitory effect than acarbose (a drug used for the treatment of diabetes) (IC_50_: 14.72 μg mL^−1^). In AChE inhibition, the water extract of the plant (IC_50_: 2.14 μg mL^−1^) showed lower inhibition than the tacrine standard (IC_50_: 1.18 μg mL^−1^). The methanol extract of the plant showed lower inhibitory effect (IC_50_: 16.31 μg mL^−1^) than acetazolamide (IC_50_: 1.85 μg mL^−1^) on hCA II.^12^ In many studies, the effects of *Phlomis* species on enzyme inhibition and some metabolic diseases have been determined.^59^, ^60^, ^61^, ^62^

### Molecular docking analysis

The quantitative result of the docking score reveals the activity of molecules against proteins. Molecular activity rises with increasing molecule–protein interaction. The most negative value among the docking scores indicates the highest activity. Molecular docking scores of major phytochemicals in *P. capitata* with enzymes are given in Table 8. The molecular interactions of the cyranoside phytochemical with the highest docking score against enzymes are given in Table 9.

**Table 8:** Docking scores of the AChE, BChE, *α*‐glycosidase, *α*‐amylase, and hCA I and hCA II enzymes with the major phenolic compounds of *Phlomis capitata*

Complex	Docking scores (kcal mol^−1^)	Complex	Docking scores (kcal mol^−1^)	Complex	Docking scores (kcal mol^−1^)
AChE (4M0E) Cyranoside	**−11.2**	BChE (4BDS) Cyranoside	**−10.0**	*α*‐amylase (1DHK) Cyranoside	**−8.2**
AChE (4M0E) Chlorogenic acid	−8.0	BChE (4BDS) Chlorogenic acid	−8.4	*α*‐amylase (1DHK) Chlorogenic acid	−6.7
AChE (4M0E) Naringenin	−8.7	BChE (4BDS) Naringenin	−8.6	*α*‐amylase (1DHK) Naringenin	−7.1
AChE (4M0E) Quinic acid	−6.6	BChE (4BDS) Quinic acid	−6.1	*α*‐amylase (1DHK) Quinic acid	−5.9
AChE (4M0E) Vanilic acid	−6.6	BChE (4BDS) Vanilic acid	−6.0	*α*‐amylase (1DHK) Vanilic acid	−6.0
AChE (4M0E) Tacrine	−7.4	BChE (4BDS) Tacrine	−8.1	*α*‐amylase (1DHK) Acarbose	−7.8
*α*‐glycosidase (3WY1) Cyranoside	**−10.3**	Carbonic anhydrase I (1AZM) Cyranoside	**−8.7**	Carbonic anhydrase II (3HS4) Cyranoside	**−8.3**
*α*‐glycosidase (3WY1) Chlorogenic acid	−8.4	Carbonic anhydrase I (1AZM) Chlorogenic acid	−7.4	Carbonic anhydrase II (3HS4) Chlorogenic acid	−7.2
*α*‐glycosidase (3WY1) Naringenin	−7.4	Carbonic anhydrase I (1AZM) Naringenin	−8.0	Carbonic anhydrase II (3HS4) Naringenin	−7.6
*α*‐glycosidase (3WY1) Quinic acid	−6.7	Carbonic anhydrase I (1AZM) Quinic acid	−6.1	Carbonıc anhydrase II (3HS4) Quinic acid	−5.9
*α*‐glycosidase (3WY1) Vanilic acid	−6.1	Carbonic anhydrase I (1AZM) Vanilic acid	−5.9	Carbonıc anhydrase II (3HS4) Vanilic acid	−6.3
*α*‐glycosidase (3WY1) Acarbose	−9.8	Carbonic anhydrase I (1AZM) Azetazolamide	−6.0	Carbonic anhydrase II (3HS4) Acetazolamide	−6.6

*Note*: Values in bold are those with the highest molecular docking scores.

**Table 9:** Molecular interactions of AChE, BChE, *α*‐glycosidase, *α*‐amylase, and hCA I and hCA II enzymes with cyranoside of *Phlomis capitata*

Complex	Types of interactions	Interacting residues
AChE (4M0E)	Hydrogen bonding	Gln1837D, Gln1837D, Met1846D, Ala1853D, Ala1853D, Ala2002D, Ala2004D, Val2005D, His2006D, Leu2231D, Asn2275D
	Hydrophobic interactions	Met1846D, Trp1850D, Trp1850D
	π bonding	His2230D
BChE (4BDS)	Hydrogen bonding	Asn65A, Asp67A, Gly112A, Gly112A, Glu194A, Ser284A, Ser284A
	Hydrophobic interactions	Asp67A, Trp79A
	π bonding	Trp79A
*α*‐Amylase (1DHK)	Hydrogen bonding	Asn65A, Asp67A, Gly112A, Gly112A, Glu194A, Ser284A, Ser284A
	Hydrophobic interactions	Asp67A, Trp79A
	π bonding	Trp79A
*α*‐Glycosidase (3WY1)	Hydrogen bonding	Arg434A, Arg434A, Gln435A, Asp438A, Ser576B, Asn578B, Asp972B, Phe987B
	π bonding	His345A
	Salt bridges	His345A
Carbonic anhydrase I (1AZM)	Hydrogen bonding	Asp2A, Trp3A, Trp3A, Asn9A, His62A, Pro238A, Gln240A, His241A
	Hydrophobic interactions	His62A
	Salt bridges	Lys168A
Carbonic anhydrase II (3HS4)	Hydrogen bonding	Tyr4A, Tyr4A, His61A, Ser96A, Ser96A, Gln100A, Glu235A
	Hydrophobic interactions	Phe227A, Phe227A, Phe227A

Cyranoside, chlorogenic acid, naringenin, quinic acid and vanilic acid were identified as the major bioactive components in the plant extract. Molecular docking studies were carried out to asses the binding affinity of these compounds with key enzymes, specifically AChE [Protein Data Bank (PDB) ID: 4M0E], BChE (PDB ID: 4BDS), *α*‐glucosidase (PDB ID: 3WY1), *α*‐amylase (PDB ID: 1DHK), hCA I (PDB ID: 1AZM) and hCA II (PDB ID: 3HS4). Among the compounds tested, cyranoside demonstrated the most favorable binding interactions across all target enzymes. The docking studies demonstrated that the optimal binding interaction for cyranoside was achieved with AChE, exhibiting the highest binding affinity, with a docking score of −11.2 kcal mol^−1^. Subsequent docking scores for cyranoside interactions with BChE, *α*‐glucosidase, *α*‐amylase, and hCA I and hCA II enzymes were calculated as −10.0, −10.3, −8.2 and −8.3 kcal mol^−1^, respectively. After the studies, the binding modes were analyzed, and it was determined that cyranoside showed the highest binding affinity of all enzymes according to the docking scores (Table 9). Molecular interactions are presented in Table 9.

**Figure 5:**
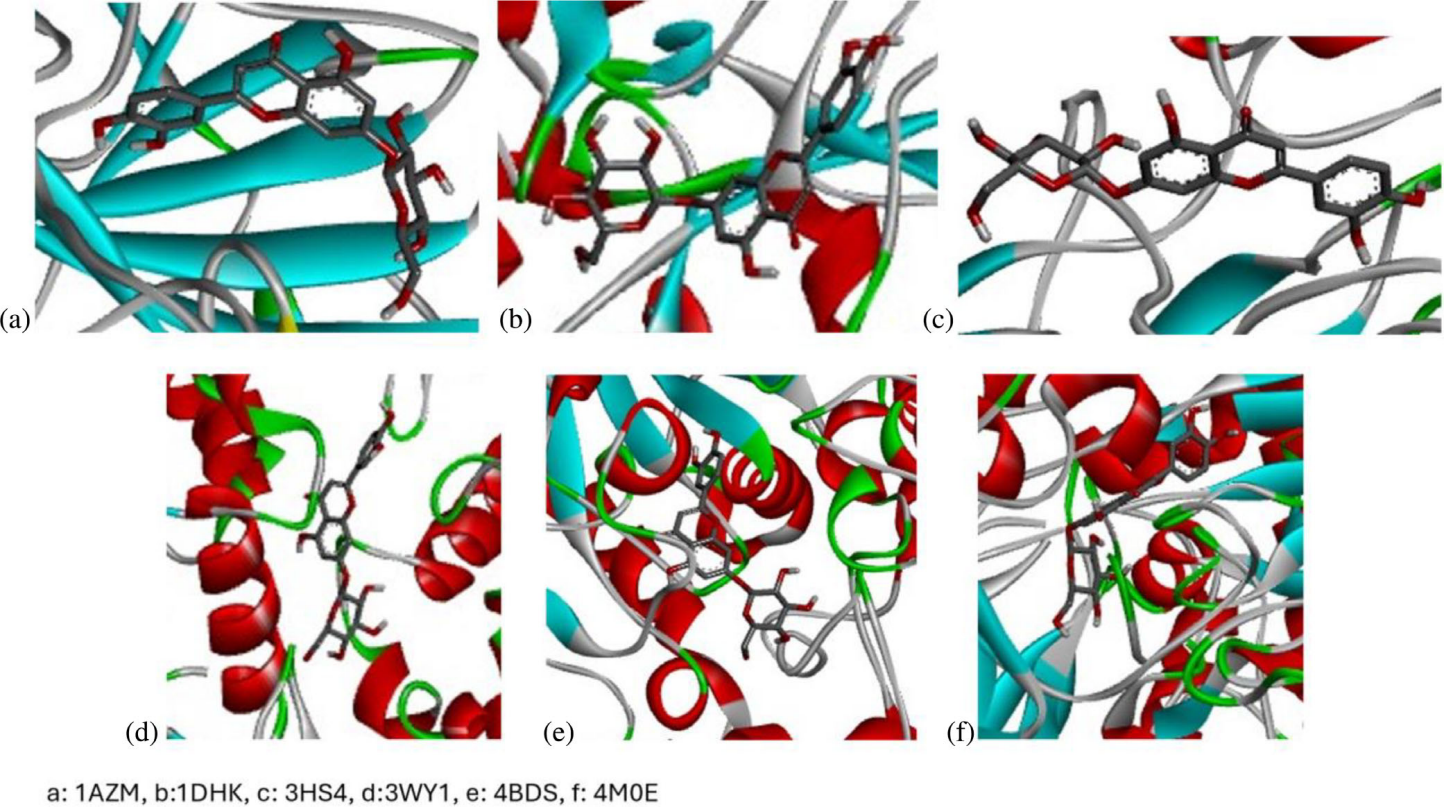
The 2D binding mode of cyranoside ligand in hCA I (a), *α*‐amylase (b), hCA II (c), *α*‐glycosidase (d), BChE (e) and AChE (f)

Following identification of docking scores, a detailed analysis of the binding modes was performed to evaluate molecular interactions such as hydrogen bonding, hydrophobic interactions and π‐π stacking. The binding affinity of bioactive molecules was supported by these interactions, suggesting a potential for inhibitory effects on these enzymes. The molecular interactions of compounds with the enzyme active sites are illustrated in Fig. 5. Chemical interactions between molecules and proteins typically involve hydrogen bonds, π‐π, halogen bonds, and polar and hydrophobic interactions. The 2D—3D structures of conventional hydrogen bond, Pi‐Pi‐shaped, Pi‐alkyl, Pi‐anion, carbon hydrogen bond, Pi‐pi stacked and Pi‐sulfur bonds are shown in Fig. 5 (see also Tables 10 and 11).

**Table 10:** The 3D binding mode of cyranoside ligand in hCA I (a), *α*‐amylase (b), hCA II (c), *α*‐glycosidase (d), BChE (e) and AChE (f)

The 3D binding mode of cyranoside	
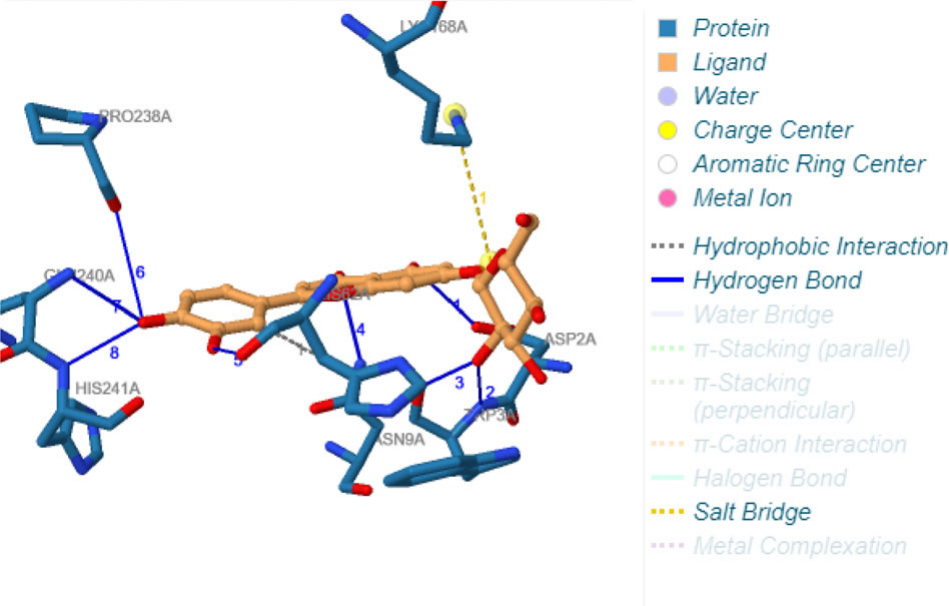	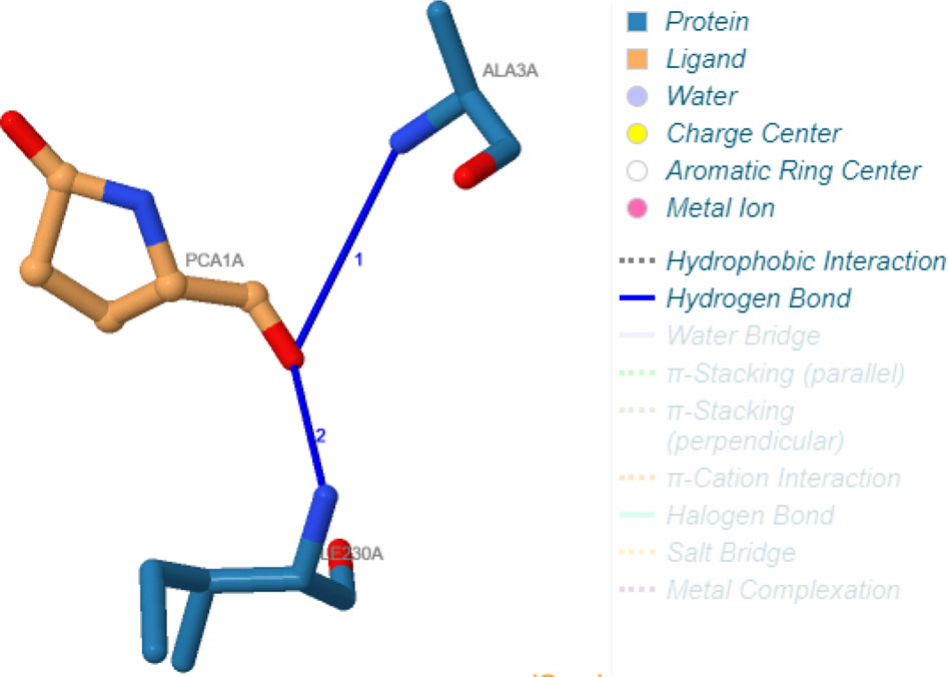
1AZM	b 1DHK
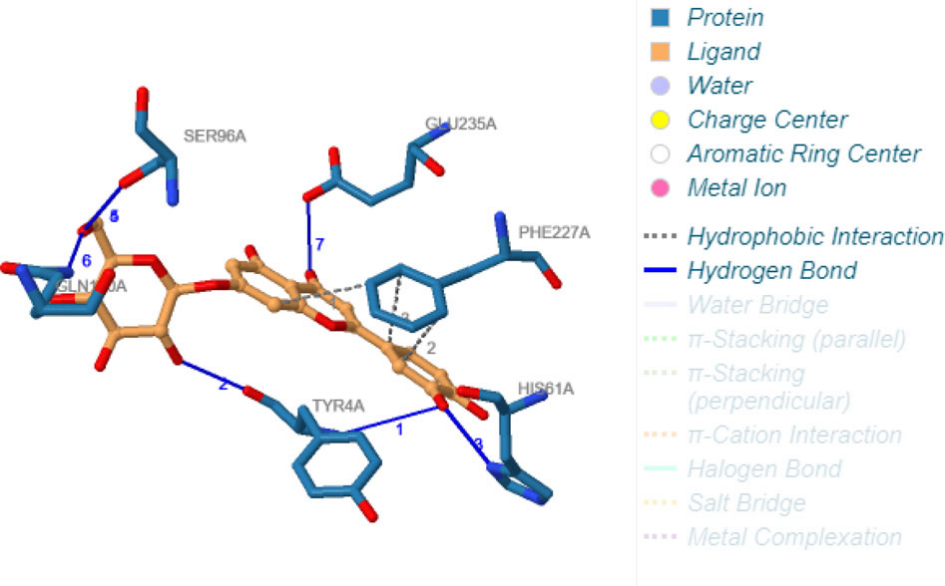	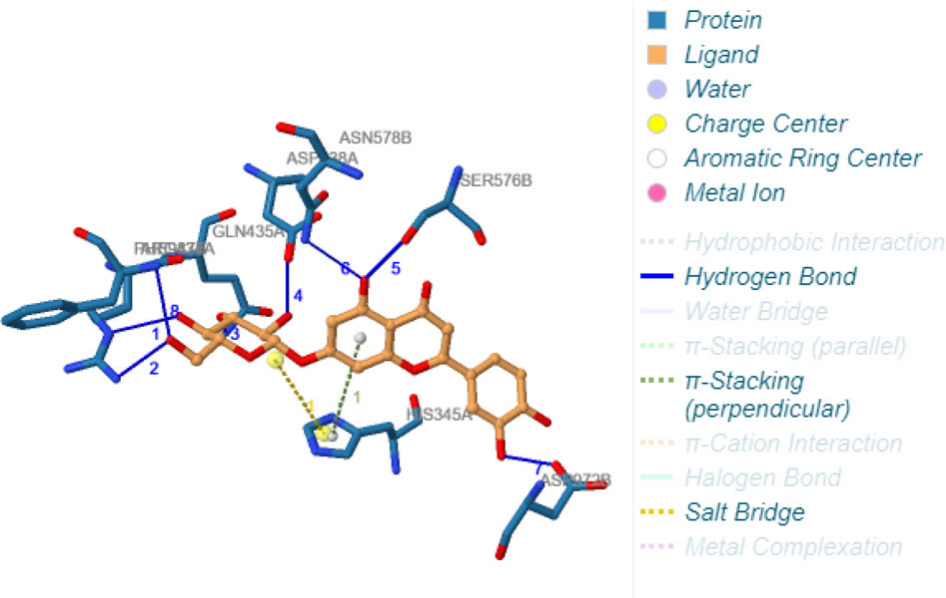
c 3HS4	d 3WY1
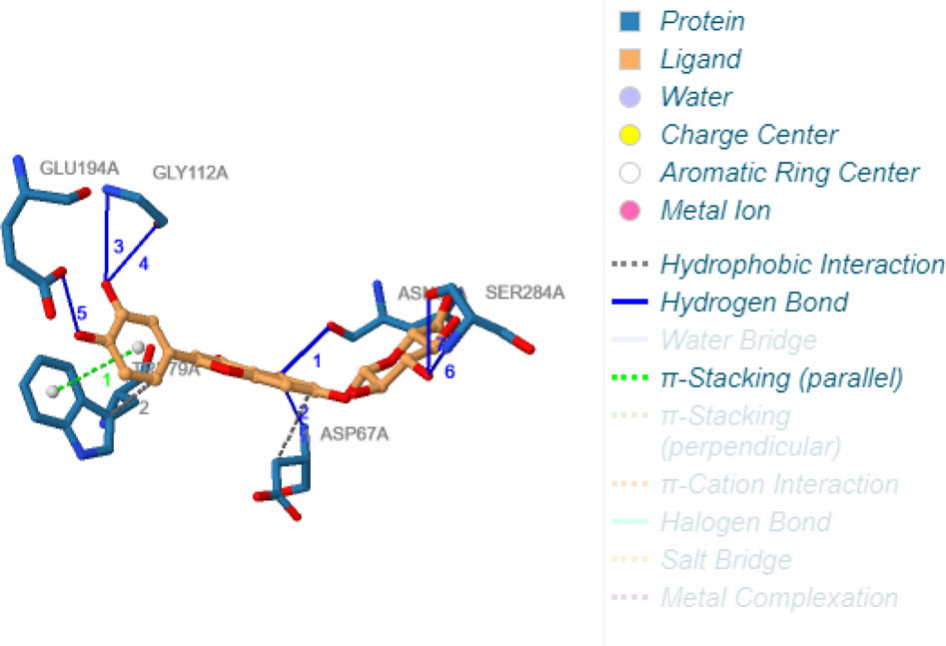	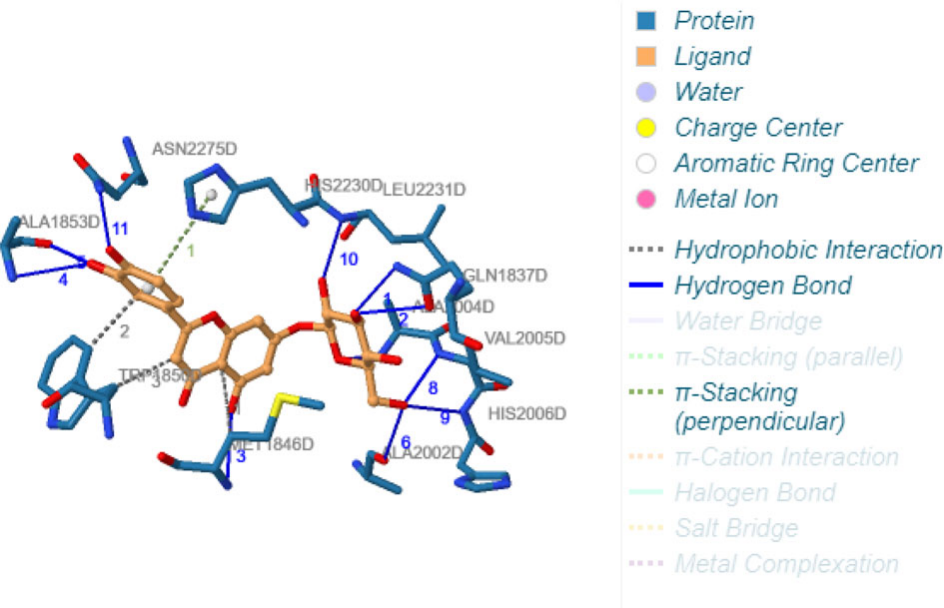
e 4BDS	f 4M0E

**Table 11:** The H bonding mode of cyranoside ligands in hCA I (a), *α*‐amylase (b), hCA II (c), *α*‐glycosidase (d), BChE (e) and AChE (f)

The hydrogen bonding mode of cyranoside	
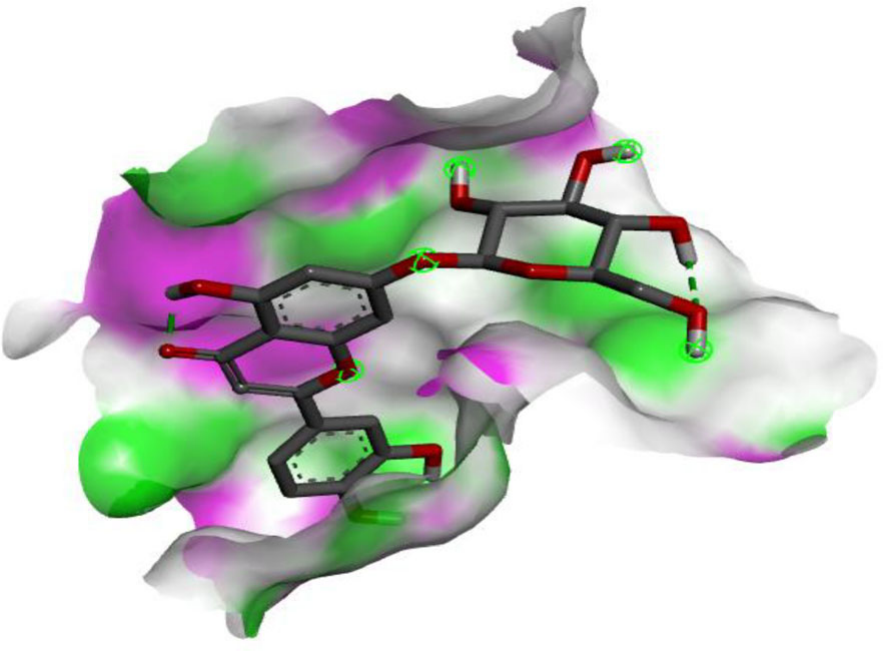	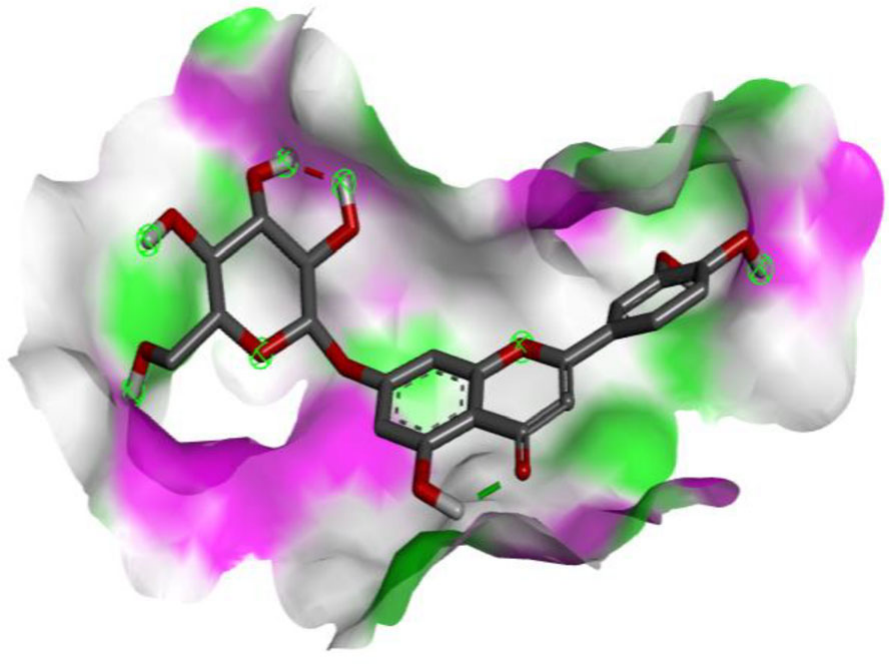
1AZM	b 1DHK
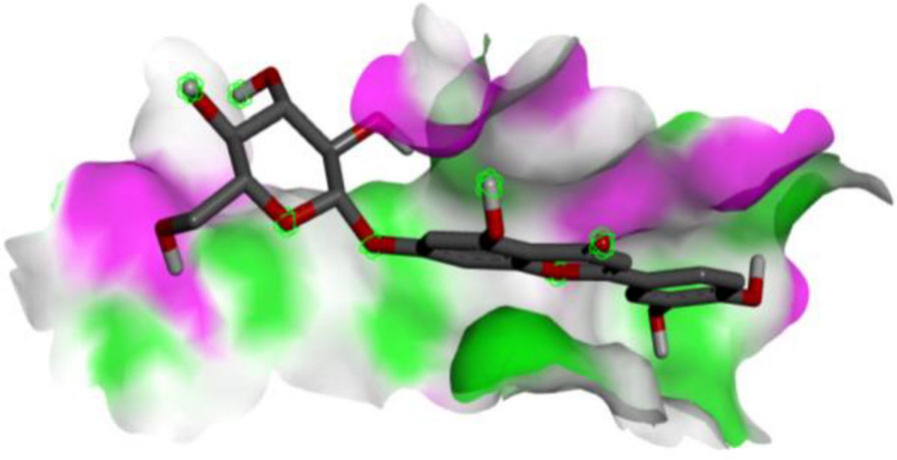	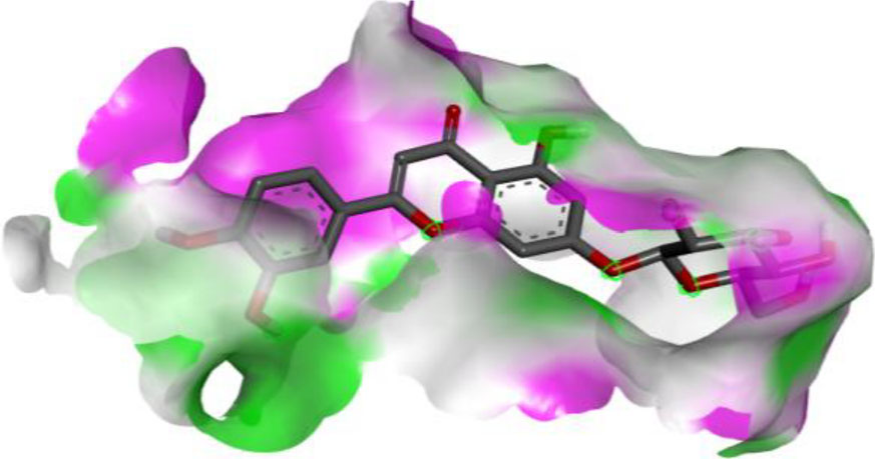
c 3HS4	d 3WY1
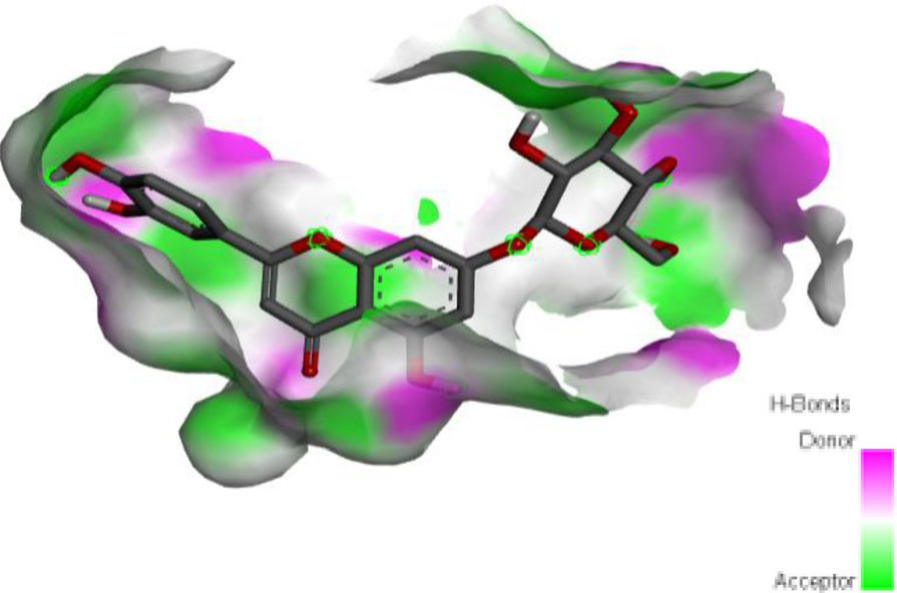	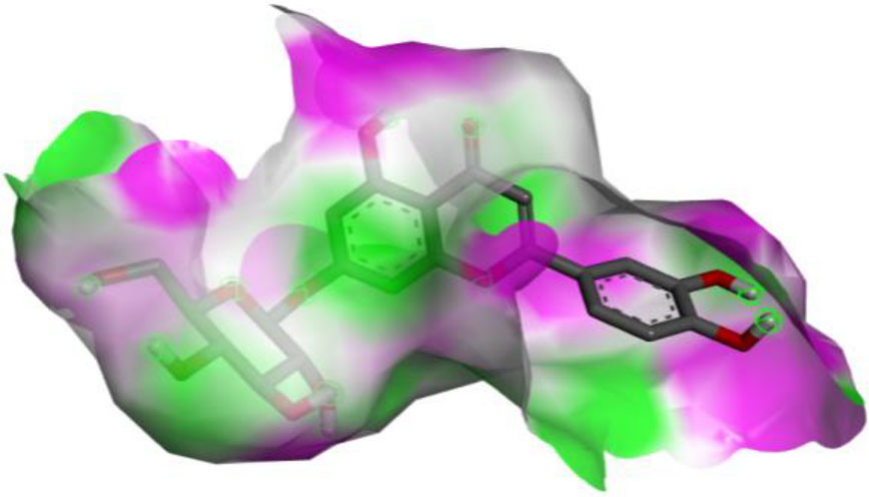
e 4BDS	f 4M0E

In the enzyme kinetics findings, the inhibitory activities of AChE and BChE by the ethanol extract of the plant were determined as 3.26 and 7.15 (IC_50_ (μg mL^−1^), respectively. In parallel, molecular docking studies revealed that cyranoside revealed docking scores of −11.2 kcal mol^−1^ for AChE and −10.0 kcal mol^−1^ for BChE. The docking score values for tacrine used as a standard were calculated as −7.4 and −8.1 kcal mol^−1^, respectively. These findings suggest a strong correlation between the experimental and computational data, indicating that both methods are consistent and mutually supportive. Notably, cyranoside demonstrated a higher binding affinity, as indicated by its more favorable docking scores, compared to tacrine, which is commonly used as a reference inhibitor of cholinesterase enzymes. This suggests that cyranoside may possess superior inhibitory potential against both AChE and BChE in comparison to tacrine. A similar trend was observed in the docking results for *α*‐glucosidase, *α*‐amylase, and hCA I and hCA II enzymes where the selected bioactive compounds, including cyranoside, showed significant interactions. The molecular docking analysis revealed that these compounds formed hydrogen bonds and other stabilizing interactions with critical amino acid residues within the active sites of the target proteins, further supporting their potential enzyme inhibitory activities.

## CONCLUSIONS

*Phlomis* species are important medicinal aromatic plants that are consumed as tea and are the subject of many different scientific studies. In the present study, the comprehensive phytochemical content of endemic *P. capitata* was determined by LC‐MS/MS and its deep biological activities such as antioxidant, antidiabetic, antiepilepsy, antiglaucoma and anti‐Alzeheimer properties were determined for the first time. As a result of 53 phytochemical screenings of *P. capitata*, it was determined that it contains 19 important bioactive components. The enzyme inhibitory effects of *P. capitata* on *α*‐amylase, *α*‐glucosidase, AChE, BchE, and human hCA I and hCA II highlight its potential as a valuable therapeutic agent. The molecular docking studies identified acacetin as the most potent inhibitor among the tested compounds, demonstrating strong binding affinities to key enzymes, particularly AChE and BChE, with docking scores surpassing those of the standard inhibitor, tacrine. These findings, as supported by molecular interactions such as hydrogen bonding and hydrophobic interactions, align with experimental enzyme kinetics, indicating the superior inhibitory potential of acacetin. Similar inhibitory trends were observed for *α*‐glucosidase and *α*‐amylase, further suggesting the potential of the bioactive compounds as effective enzyme inhibitors. Further research is needed to isolate and characterize the specific bioactive compounds responsible for these inhibitory effects, as well as to evaluate their efficacy in clinical settings.

## AUTHOR CONTRIBUTIONS

Eİ designed the study and established the methodology. He also performed all of the analysis and wrote the article.

## Data Availability

The data that support the findings of this study are available on request from the corresponding author.
